# Intravascular cutaneous squamous cell carcinoma (cSCC): A rare histopathologic finding with potential prognostic significance

**DOI:** 10.1016/j.jdcr.2025.08.027

**Published:** 2025-09-05

**Authors:** Alexa S. Podolsky, Rachel Manci, Samantha S. Sattler, Daniel Lozeau, Jordan B. Slutsky

**Affiliations:** a Renaissance School of Medicine, Stony Brook University, Stony Brook, New York; b Department of Dermatology, Stony Brook University, Stony Brook, New York

**Keywords:** cutaneous squamous cell carcinoma, high-risk, intravascular invasion

## Introduction

Cutaneous squamous cell carcinoma (cSCC) is the second most common cutaneous malignancy, and its incidence continues to increase over time. While cSCC generally portends a favorable prognosis, significant morbidity and mortality are possible in its more advanced stages.^1^ The high-risk prognostic factors currently described by the American Joint Committee on Cancer (AJCC) eighth edition and the Brigham and Women’s Hospital (BWH) include tumor diameter greater than 2-cm, depth of invasion beyond the subcutaneous fat, bony invasion, poor-differentiation, and perineural invasion.^2^ Intravascular invasion of cSCC is a rare histopathologic finding that is not currently described in either cSCC staging guideline, yet may influence locoregional recurrence and patient prognosis.^3^, ^4^, ^5^ Herein, we present 2 cases of cSCC with intravascular invasion identified during Mohs micrographic surgery (MMS), summarize the prognostic data that are available to date, and provide management recommendations for surgical cases exhibiting this high-risk feature.

## Case reports

### Case 1

**Fig 1:**
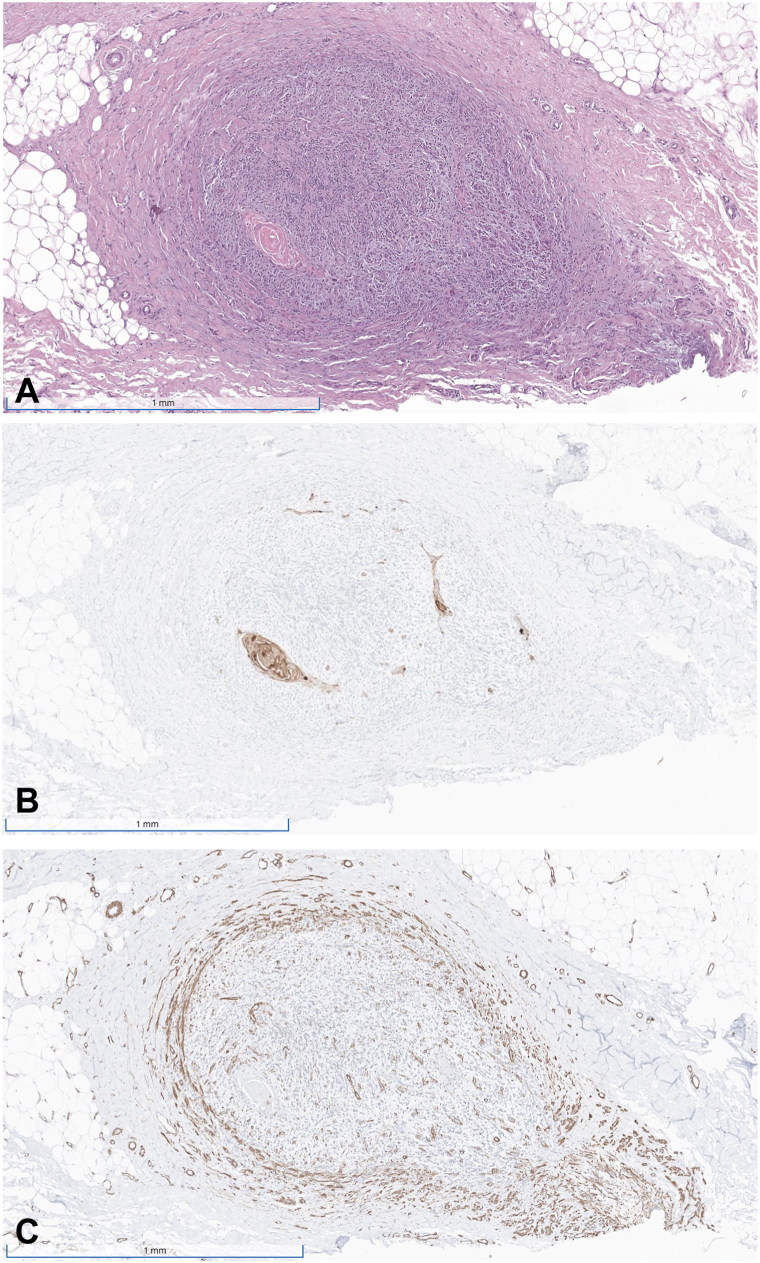
H&E staining of cutaneous squamous cell carcinoma within a large-caliber arteriole **(A)**. CK5/6 immunohistochemical stain highlights the carcinoma within the vessel **(B)**, while SMA accentuates the smooth muscle in the vessel wall **(C)**.

A 62-year-old male with history of renal transplantation 5 years prior, on 4 mg tacrolimus daily and 360 mg mycophenolate mofetil twice daily, was referred for MMS of a biopsy-proven well-differentiated cSCC presenting as a 3-cm keratotic nodule on the left vertex scalp. Medical history was negative for previous skin cancers. MMS was performed with the first stage revealing cSCC invasion beyond the subcutaneous fat, perineural invasion involving a nerve less than 0.1 mm in diameter, and intravascular cSCC obstructing a large-caliber arteriole (Fig 1). Negative margins were achieved after 2 Mohs stages, and the patient was referred to oncology for further evaluation, given that the tumor was upstaged from an initial BWH T2a/AJCC T2 to BWH T2b/AJCC T3.

Magnetic resonance imaging (MRI) of the neck was obtained, which revealed no evidence of metastatic disease. Radiation oncology recommended dermal brachytherapy to the prior surgical site to decrease the risk of local recurrence. The patient received a dose of 36-Gy in 6 weekly fractions, with no evidence of recurrence noted by 24-month follow-up.

### Case 2

**Fig 2:**
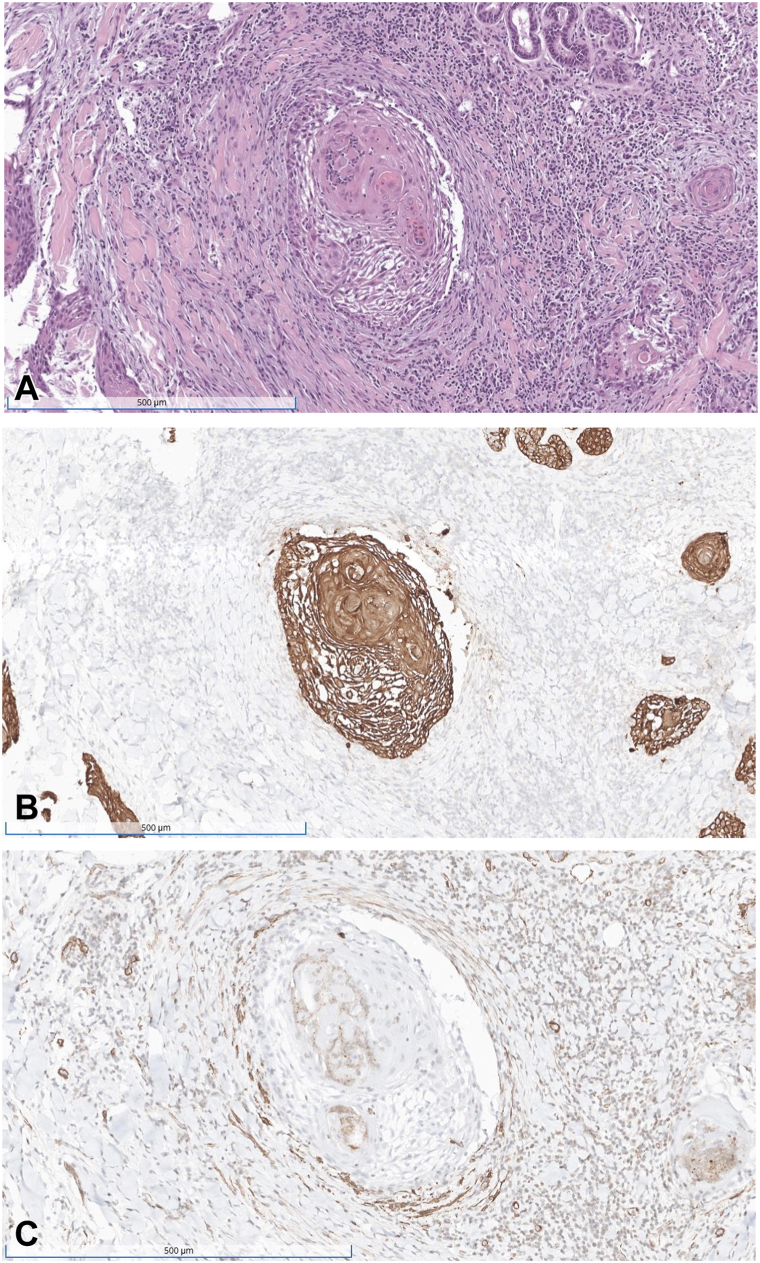
H&E staining of cutaneous squamous cell carcinoma around as well as within an arteriole **(A)**. CK5/6 stain confirms the presence of carcinoma within the vessel wall **(B)**, with SMA again highlighting the smooth muscle within the vessel wall **(C)**.

A 71-year-old male with history of melanoma-in-situ was referred for MMS of a biopsy-proven cSCC in-situ presenting as a 2-cm excoriated keratotic nodule on the left medial frontal hairline. MMS was performed with the first stage revealing a well-differentiated cSCC with invasion beyond the subcutaneous fat without perineural invasion. Intravascular cSCC within an arteriole was also noted (Fig 2). Negative margins were achieved after 2 Mohs stages, and the patient was referred to oncology for additional management, given the tumor was upstaged from initial BWH T0/AJCC Tis to BWH T2b/AJCC T2.

Computed tomography (CT) of the neck and chest revealed no evidence of metastatic disease. CT of the abdomen revealed an indeterminate peritoneal nodule, which resolved by the time of a later positron emission tomography (PET) scan. Radiation oncology recommended adjuvant dermal brachytherapy to the prior surgical site. The patient received a dose of 36 Gy in 6 weekly fractions, with no evidence of recurrence noted by 4-month follow-up.

## Discussion

Intravascular cSCC is a rare, potentially high-risk phenomenon, and its clinical implications have not been previously defined. To our knowledge, there have been only 3 other case reports in the literature to date where intravascular cSCC was identified at the time of initial treatment (Table I). Of these cases, 2 were treated with MMS followed by adjuvant radiation therapy and 1 was treated with MMS alone. Of the total 5 cases of intravascular cSCC, including the 2 reported herein, all had at least 1 high-risk BWH/AJCC feature in addition to intravascular invasion.^3^, ^4^, ^5^ Of note, 2 of these 5 patients had no prior medical history and were not on immunosuppression, highlighting that this feature can be present in the general population. One recurrence was identified at 17-month follow-up as a nodule arising in the prior radiation site of a patient who had been treated with MMS followed by adjuvant radiation therapy.^3^ The recurrent tumor exhibited invasion into a 0.12-mm-diameter nerve and clear margins were achieved with repeat MMS.

**Table I:** Literature review of intravascular cSCC cases

Article title	Authors	Article type	Patient age/sex	Relevant patient comorbidities	cSCC location	cSCC high-risk features	Treatment	Outcome
High-risk cutaneous squamous cell carcinoma with intravascular invasion in a patient with systemic sclerosis	Hoverson K, Heard MA, Lezanski-Gujda A, Evans TR, Lacket JN	Case report	70s/F	Systemic sclerosis (on 3000 mg mycophenolate mofetil daily)	Scalp	Poor-differentiation, invasion beyond subcutaneous fat, size >2 cm, perineural invasion	Mohs surgery followed by adjuvant radiation therapy (50 Gy in 20 fractions)	Recurrence at 17 mo, treated with additional Mohs surgery
Intravascular involvement of cutaneous squamous cell carcinoma	Tripathi SV, Council ML	Case report	72/M	B-cell lymphoma, in remission, s/p chemotherapy 10-y prior	Left forehead	Moderate differentiation, invasion beyond subcutaneous fat, size >2 cm	Mohs surgery followed by adjuvant radiation therapy (6600 cGy in 33 fractions of 200 cGy)	No recurrence at 12 mo
Squamous cell carcinoma tumor thrombus encountered during Mohs micrographic surgery	Mendese G, Bordeaux J, Pattee S, Maloney M	Case report	83/F	None	Right forearm	Size >2 cm	Mohs surgery	No recurrence at 21 mo
Intravascular squamous cell carcinoma treated with cemiplimab	Rose AN, Yilmaz E, Durken JR	Case report	64/M	None	Left cheek	Unknown	Initial lesion treated with excision and radiation; recurrence with intravascular invasion treated with cemiplimab	Intravascular invasion was identified at time of recurrence, which was treated with cemiplimab
This report—case #1			62/M	Renal transplantation 5-y prior (on 4 mg tacrolimus and 720 mg mycophenolate mofetil daily)	Left vertex scalp	Invasion beyond subcutaneous fat, size >2 cm	Mohs surgery followed by adjuvant radiation therapy	No recurrence at 24 mo
This report—case #2			71/M	None	Left medial frontal hairline	Invasion beyond subcutaneous fat, size >2 cm	Mohs surgery followed by adjuvant radiation therapy	No recurrence at 4 mo

One additional case of intravascular cSCC has been reported, where invasion was detected upon tumor recurrence rather than during initial treatment with excision and radiation. (Table I).^6^ This patient was successfully treated with 10 months of cemiplimab.^6^

This histopathologic phenomenon was also commented on in a retrospective review of head and neck cSCC performed by Yaqoob et al (2024).^1^ These authors attempted to identify cSCC high-risk features, and of the 1197 cSCCs involved in this analysis, 2 of 5 cases with intravascular invasion developed locoregional recurrence, which was determined to be a statistically significant high-risk feature.^1^ Moreover, numerous reports have indicated that cSCC satellitosis and in-transit metastases (S-ITM) increase the risk of tumor recurrence and disease-specific death, with overall outcomes similar to those with lymph node positivity.^7^, ^8^, ^9^, ^10^ Intravascular cSCC may represent the initial step of S-ITM, demonstrating the earliest recognizable sign that malignant cells have left the primary tumor bed. Therefore, these findings should be considered high-risk features and their incorporation into clinical staging systems should be considered.

Taken together, dermal vascular occlusion by cSCC may suggest a more aggressive phenotype, which should be considered a risk factor for potentially worsened prognosis. The reported cases did not exhibit a worse prognosis likely due to their prompt treatment with surgery and radiation therapy. Inclusion of this finding in formal cSCC guidelines may thus be warranted to ensure such prompt treatment. An interdisciplinary approach with hematology-oncology and radiation oncology as well as baseline imaging and radiation may be indicated, even in the absence of other high-risk features. Future studies are warranted to quantify the direct impact of intravascular cSCC on prognosis.

## Conflicts of interest

None disclosed.
